# Water phase distribution and its dependence on internal structure in soaking maize kernels: a study using low-field nuclear magnetic resonance and X-ray micro-computed tomography

**DOI:** 10.3389/fpls.2024.1529514

**Published:** 2025-01-24

**Authors:** Baiyan Wang, Shenghao Gu, Juan Wang, Guangtao Wang, Xinyu Guo, Chunjiang Zhao

**Affiliations:** ^1^ Nanjing Agricultural University, MSU Institute, Nanjing, China; ^2^ Beijing Key Lab of Digital Plant, Information Technology Research Center, Beijing Academy of Agriculture and Forestry Sciences, Beijing, China

**Keywords:** phenotyping, hydration, water absorption, seed emergence, kernel moisture

## Abstract

**Introduction:**

The formation of yield and quality in maize involves the accumulation of substances such as starch, proteins, and fats, which interact with water within the kernel. Although temporal dynamics of grain moisture and its functional and environmental determinants have been broadly demonstrated, we still do not have a comprehensive understanding of the distribution of water phase within a kernel.

**Methods:**

We investigated the relationship between tissue structural traits, including embryo volume (EMBV), endosperm volume (ENDV), vitreous endosperm volume (VEV), floury endosperm volume (FEV), and water content in different phases, such as bound water, semi-bound water, and free water, in maize kernels under different cultivars, nitrogen application rates, and soaking durations by combining low-field nuclear magnetic resonance (LF-NMR) and X-ray microcomputed tomography (μ-CT) for kernels.

**Results:**

The results demonstrate that bound water is the major phase (57-82%) in maize kernels, and this proportion decreases with prolonged soaking duration. The bound water content and semi-bound water content positively correlate to ENDV, VEV, and EMBV, whereas free water content correlates to ENDV, EMBV, and VEV in descending order of correlation coefficient. This indicates that water might penetrate the embryo through the pedicel and vitreous endosperm through the pericarp during soaking.

**Discussion:**

Finally, we suggested that the proportion of semi-bound water could be a robust indicator to predict moisture content in maize kernels. The study provides a preliminary understanding of the structural basis of water distribution in maize kernels, thereby opening up the potential for designing efficient production systems and breeding cultivars well-suited for mechanical harvesting.

## 1 Introduction

Maize (*Zea mays L.*), a paramount grain crop globally, is essential for maintaining food and nutritional security worldwide. The Sustainable Development Goals (SDGs) seek to eliminate global hunger while simultaneously prioritizing year-round access to secure and nutritious food (Katila et al., 2019). Accurate regulation of kernel moisture in maize is crucial for both efficiently mechanical grain harvesting in modern agriculture production (Zhang et al., 2023), and for maximizing yield, quality, and efficiency in post-harvest processes (Li et al., 2021). Consequently, comprehending the factors influencing grain moisture and the associated mechanisms is a critical for designing efficient production systems and breeding appropriate cultivars for mechanical harvesting.

Rapid germination, vigorous seedling, and uniform emergence are decisive factors for a high-yielding cropping system (Egli and Rucker, 2012; Rehman et al., 2015). Soaking kernels in water is one of the best strategies to promote seed germination and enhance seedling vigor by softening the seed coat and breaking seed dormancy especially for small-scale farmers (Ahmed and Mahdy, 2022; Ashraf and Foolad, 2005; Harris et al., 1999; Sanatawy et al., 2021). Water uptake in maize seeds is influenced by the structure and chemical components in kernels (McDonald et al., 1994). Various factors significantly influence the internal structure of maize kernels. For example, nitrogen availability affects starch accumulation and endosperm composition (Feng et al., 2024), while drought and high temperatures influence kernel size and internal density (Niu et al., 2021). Additionally, different cultivars exhibit notable differences in pericarp permeability and endosperm type (Zhang and Xu, 2019). However, water uptake patterns in maize kernels have been rarely investigated.

Water can be categorized into several phases: free water, semi-bound water, and bound water, depending on its functions and interactions within biological systems (Almeida et al., 2007; Khan et al., 2017). Free water can traverse cell membranes and evaporate at ambient temperature, which is essential for metabolic transport and thermal regulation in organisms (Wang et al., 2019; Li et al., 2023). Conversely, bound water is closely connected with biomolecules, including proteins, polysaccharides, and many biological constituents. This phase hardly engages with the environment but contributes to the stabilization of biomolecular structures (Dargaville and Hutmacher, 2022). Semi-bound water is present on the surface of plant colloids, exhibiting relatively modest adsorption forces, which allows for some fluidity while being less prone to evaporation than free water.

Recent study breakthroughs have advanced water content assessment from a singular measurement to a comprehensive evaluation of water phases. Conventional techniques, such as drying, microwave resonators, and near-infrared spectroscopy, have demonstrated efficacy in quantifying individual water content (Kraszewski et al., 1991; Watanabe et al., 2012; ISO, 2009). Low-field nuclear magnetic resonance (LF-NMR) is an innovative non-destructive detection technology that measures the mobility of hydrogen protons via relaxation time assessments, widely applied in various fields, including food (Zhang et al., 2021), agriculture (Qiao et al., 2021), medicine (Zhang et al., 2023), and polymer materials (Hou et al., 2023). This facilitates the observation of variations in water content, water phases, and fat content in samples (Song et al., 2022). Nuclear magnetic resonance (NMR) relaxation technology has demonstrated considerable advantages in investigating the rheological properties of polymers, kernel viability, and the water characteristics of materials (Krishnan et al., 2004; Manz et al., 2005; Dekkers et al., 2016). Both NMR and LF-NMR have been used to study water phase distribution in cereal grains. For example, Krishnan et al. (2014) found that nitrogen application increased bound and semi-bound water while reducing free water in maize kernels, though the role of internal morphological structure was not addressed. Zhao et al. (2018) developed a method using *T_2_* relaxation profiles from LF-NMR to measure water content in individual kernels. Yang et al. (2019) used LF-NMR to analyze water phase distribution and rice kernel vigor under salt stress. Chen et al. (2020) investigated dehydration and water absorption dynamics in maize kernels, while Li et al. (2022) applied imaging to assess water status in maize ears and kernels, proposing new approaches for grain phenotyping. However, the spatial distribution of water phases and their relationship with kernel internal structure remains unclear.

**Figure 1:**
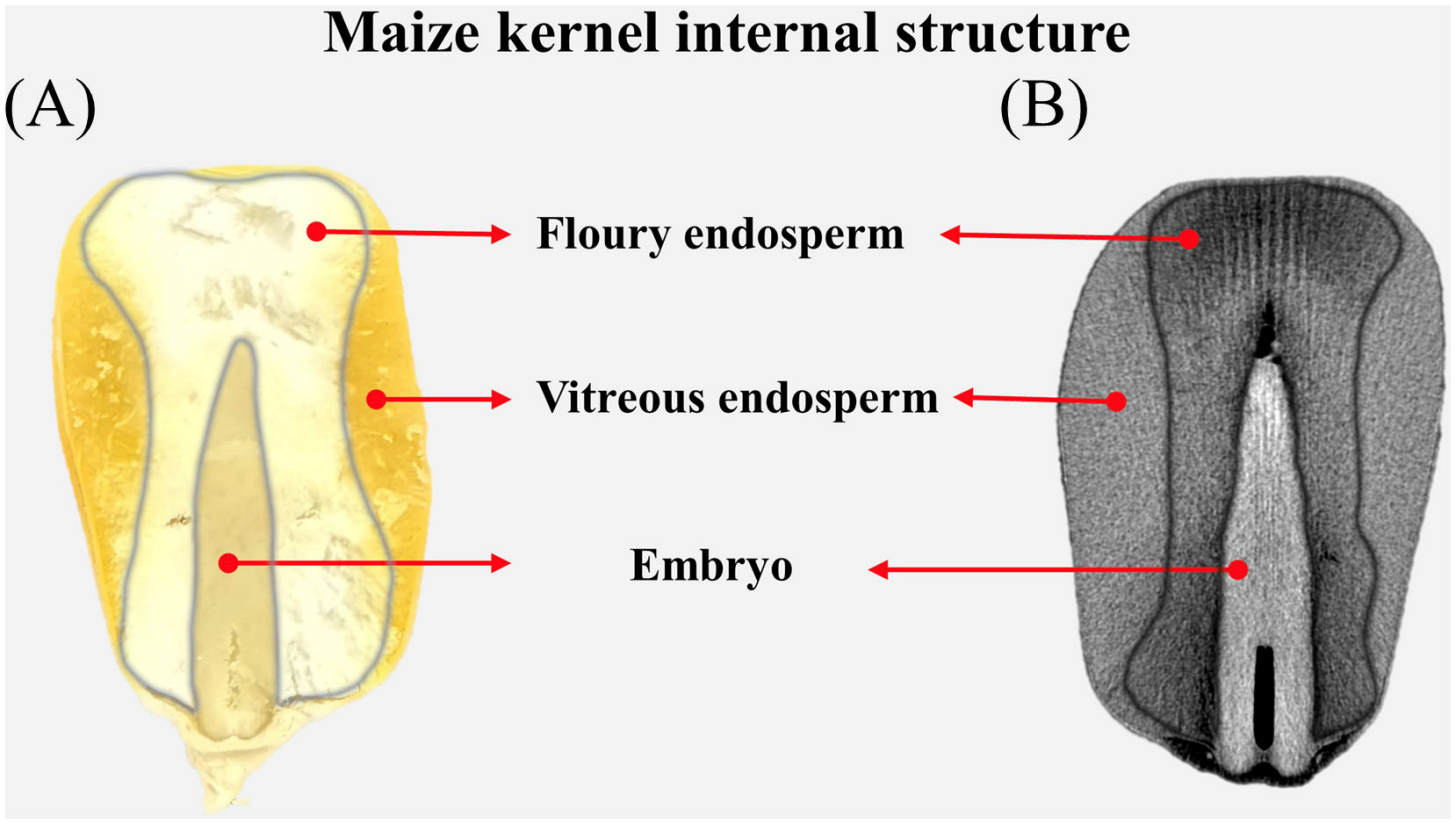
Internal structure of the maize kernel viewing from a real image **(A)** and a μ-CT scan **(B)**.

From a structural perspective, maize kernels consist of three primary components: the embryo, endosperm, and pericarp (**Figure 1**). The endosperm, comprising the bulk of the kernel’s mass, is categorized into vitreous and floury endosperm for the storage of proteins and starch (Nuss and Tanumihardjo, 2010; Xu et al., 2019a). X-ray micro-computed tomography (μ-CT), a non-destructive technique for examining the interior structures of samples, has been utilized to extract micro-phenotypic indicators and investigate the variations among various kernel types across different species (Hou et al., 2019; Le et al., 2019; Dong et al., 2020; Crozier et al., 2022). Dong et al. (2020) further found a correlation between the kernel breaking rate and micro-structural parameters in maize kernels. Liao et al. (2022) elucidated the spatial-temporal distribution of the endosperm cavity in maize kernels and identified candidate genes potentially associated with endosperm development. The μ-CT, offering enhanced resolution for imaging the interior structure of grains, may serve as a potent and complementary tool to LF-NMR in identifying various water phases and their dynamics within grains. The effectiveness of utilizing LF-NMR and μ-CT to investigate the temporal-spatial distribution of water phases within maize kernels is still uncertain.

In this study, we investigate structural traits and water content in different phases in maize kernels under different cultivars, nitrogen application rates, and soaking durations by combing LF-lNMR and μ-CT. We first characterize the temporal dynamics of water in different phases in soaking maize kernels. We then explore to what extent the tissue volume can explain the water penetration and distribution in maize kernel. Finally, we evaluate the reliability of water phase characteristics in predicting kernel moisture content.

## 2 Materials and methods

### 2.1 Experimental materials and preparation

The field experiment was conducted in Gongzhuling City, Northeast China (124°82′26″ E, 43°79′13″ N). Three typically and locally cultivated varieties with varying protein contents were selected (**Table 1**). The field experiment was sown on April 28, 2023 and harvested on October 2, 2023. Four nitrogen application rates were 30 kg·hm^-2^, 60 kg·hm^-2^, 180 kg·hm^-2^, and 300 kg·hm^-2^. Three ears were randomly selected per plot after harvest, from the middle of each 30 well-developed kernels were sampled. Subsequently, kernels were deactivated by drying at 105°C for 30 minutes at first, and then were dried at 80°C until constant weight was achieved. Ultimately, three kernels with uniform shape and size were randomly selected from the total of 90 for the soaking experiment.

**Table 1:** Nutritional components in different maize cultivars.

Cultivars	Crude Protein (%)	Crude Fat (%)	Crude Starch (%)	Hundred-grain Weight (g)
DK517	9.40	4.00	74.74	28.90
LY99	9.86	4.00	72.20	28.40
ZD958	9.26	4.50	72.64	29.88

The maize cultivars data were obtained from the China seed industry big data platform (http://202.127.42.47:6010/SDSite/Home/Index).

### 2.2 Experimental methods

#### 2.2.1 Experimental treatment and procedure

**Figure 2:**
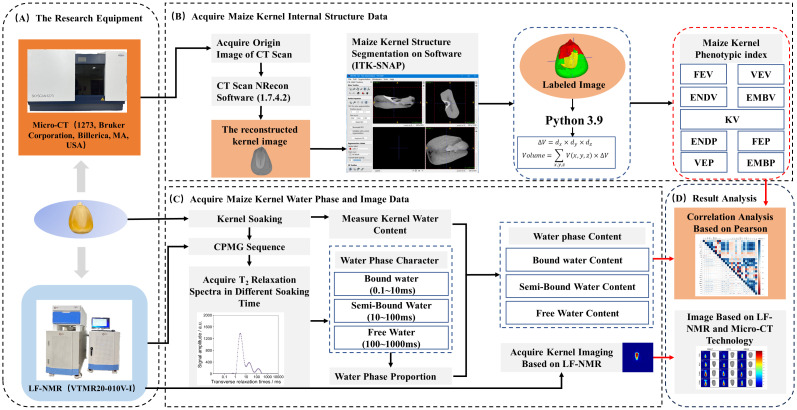
Flowchart of experimental data acquisition **(A)**, processing (**B** for Micro-CT and **C** for LF-NMR), and analysis **(D)**.

After selecting the sample, the dry weight was initially measured and recorded. Before the soaking, the maize kernels were subjected to μ-CT to acquire interior structural characteristics. To investigate the dynamic changes in water phase distribution within kernels during soaking, as well as the water phase distribution in kernels under saturated conditions, the soaking experiment was designed with five soaking durations: 1 hour (T1), 3 hours (T2), 6 hours (T3), 9 hours (T4), and 18 hours (T5). At each soaking period, the soaked kernels were extracted and delicately blotted with absorbent paper to remove surface moisture. To prevent moisture loss through evaporation, the treated kernels were promptly placed in 10 ml centrifuge tubes and then kept in a refrigerator at 4°C. Before each sample is placed on the instrument for measurement, the weight of individual kernels was measured. The kernels were subsequently placed in the LF-NMR probe for the capture of hard pulse echo signals. Furthermore, at the T5 temporal point, nuclear magnetic resonance imaging (MRI) signals of the kernels were collected. Finally, correlation analysis was used to investigate the association between water phase and interior structural morphology (**Figure 2**).

#### 2.2.2 LF-NMR detection procedure

Based on the non-invasive and non-destructive detection capabilities of LF-NMR (VTMR20-010V-I), hard pulse echo signals and nuclear magnetic resonance (NMR) imaging signals of kernels were collected at different water soaking. The parameters for kernel NMR signal acquisition were as follows: sequence option Q-CPMG, main frequency SF=20 MHz, offset frequency O1 = 393510.81 Hz, number of sampling repetitions NS=64, repetition sampling waiting time 2000 ms, number of echoes NECH=3000, echo time TE=0.2 ms, number of signal acquisition points TD=120014, 90° pulse width P1 = 6 μs, and 180° pulse width P2 = 10 μs. The acquired signal sequences were processed using the accompanying software (NIUMAG MRI Analysis Application Software) to invert the relaxation characteristics of the test samples at different water soaking times, and the fitted data were exported for subsequent analysis. The parameters for LF-NMR imaging were as follows: sequence option HSE, offset frequency 20.393 MHz, bandwidth 20 kHz, phase encoding duration 0.5 ms, repetition time (TR) 500 ms, echo time (TE) 14.2 ms, 90° pulse width 9.5 μs, 180° pulse width 14.5 μs, RG (Readout Gradient) 20 dB, PRG (Precession Readout Gradient) 3, and DRG (Data Readout Gradient) 5 dB. The collected *T_2_* relaxation inversion spectra and proton density images were named and stored for subsequent analysis.

#### 2.2.3 μ-CT scanning procedure

In this study, a μ-CT scanner (1273, Bruker Corporation, Billerica, MA, USA) was used to acquire CT phenotypic data of the maize kernels. During scanning, the kernels were fixed onto a foam support and placed in the CT scanner for imaging. The scanning parameters were set to a 3K resolution mode (3072×1944 pixels) with a pixel resolution of 15 μm. The scanning was performed at a voltage of 50 kV and a current of 200 μA, with the device executing a 180° rotational scan of the sample at 0.3° intervals.

After scanning, the raw CT scan images were reconstructed using CT Scan NRecon 1.7.4.2 software (Micro Photonics Inc, Allentown, PA, USA) to generate a sequence of high-resolution virtual cross-sectional images of the kernels (Approx. 1,695 sheets, detailed selection based on kernel size), with a resolution of 3072×1944 pixels and in 8-bit BMP format. Subsequently, these cross-sectional images were imported into the open-source software ITK-SNAP, where the floury endosperm, vitreous endosperm, embryo of the 36 kernels were precisely marked using a manual interactive labeling method. The marked images were saved in.nii format for further calculation of the 3D volumes of the floury endosperms, vitreous endosperms and embryo. The involved formulas and steps are as follows:

First, the voxel data were extracted from the.nii file, and the voxel volume ΔV can be calculated using the voxel dimensions as follows:


(1) $$\Delta V=d_{x}\times d_{y}\times d_{z}$$

where *d_x_*, *d_y_*, and *d_z_* represent the voxel dimensions in the three respective directions. The volume is then calculated by summing the volumes of all the voxels:


(2) $$Volume=\sum_{x,y,z}V\left(x,y,z\right)\times \Delta V$$

where *V(x, y, z)* represents the voxel value.

Processing marked image files, extracting voxel (i.e., three-dimensional pixel) intensity data and volume calculation were performed using Python scripts. Each voxel represents a fixed-volume spatial unit (voxel dimensions are 10 μm × 10 μm × 10 μm, corresponding to 10_-6_ mm³). Finally, the total intensity value of all voxels was quantified using the.sum() method in NumPy arrays to represent the CT phenotypic data of the kernel. The volumes of the vitreous endosperm, floury endosperm, embryo, and the entire kernel are extracted manually for each maize kernel (**Figure 1**).

### 2.3 Extraction of kernel water phase indicators and kernel phenotypic indicators

This study extracts relevant indicators from the water phases and phenotypic structure of the maize kernels. The classification of water phases in the kernels is based on the study by (Li et al., 2023), where the relaxation time of bound water ranges from approximately 0.1 to 10 ms (*T_21_*), semi-bound water from approximately 10 to 100 ms (*T_22_*), and free water from approximately 100 to 1000 ms (*T_23_*). The signal amplitudes of the relaxation inversion spectra for bound water, semi-bound water, and free water are denoted as *A1, A2*, and *A3*, respectively. *A* represents the total signal amplitude of the maize kernel, calculated as follows:


(3) A=A1+A2+A3

Previous studies have shown a significant linear relationship between the water content of maize kernels and the signal amplitude. In this study, the kernels were initially dried, and their dry weight (DW) was recorded. Following each water soaking, fresh weight (FW) of each individual kernel was measured. Water content (TWC) and moisture content (MC) were calculated as follows:


(4) TWC=FW−DW

(5) $$\text{MC}=\frac{\text{FW}-\text{DW}}{\text{FW}}$$

Based on this, the content of each water phase is calculated as follows:


(6) BWP=A1/A

(7) SBWP=A2/A

(8) FWP=A3/A

(9) BWC=TWC×BWP

(10) SBWC=TWC×SBWP

(11) FWC=TWC×FWP

where BWP, SBWP, and FWP indicate the proportions of bound, semi-bound, and free water in the kernel relative to the total water, respectively; BWC, SBWC, and FWC represent the absolute content of bound, semi-bound, and free water in the kernel, respectively. The proportions of the floury endosperm, vitreous endosperm, endosperm, and embryo volumes to the total kernel volume (FEP, VEP, ENDP, and EMBP) are calculated by the following equations:


(12) FEP=FEV/KV

(13) VEP=VEV/KV

(14) ENDP=ENDV/KV

(15) EMBP=EMBV/KV

where FEV, VEV, ENDV, and EMBV represent the volumes of the floury endosperm, vitreous endosperm, endosperm and embryo, and KV represents the total volume of the kernel.

### 2.4 Data analysis

Data processing, statistical analysis and data visualizations were performed using Microsoft Office, SPSS, and RStudio software. Multiple comparisons in this study were performed using Duncan’s SSR test, and correlation analysis was carried out using Pearson correlation coefficients. The significance level was evaluated using *p*-values, with *p*< 0.05 considered statistically significant. The calculation method for the linear correlation coefficient is as follows:


(16) $$R^{2}=\frac{\sum^{}\left(x_{i}-\bar{x}\right)\left(y_{i}-\bar{y}\right)}{\sqrt{\sum^{}{\left(x_{i}-\bar{x}\right)}^{2}\cdot \sum^{}{\left(y_{i}-\bar{y}\right)}^{2}}}$$

where $x_{i}\;$ and $y_{i}$ represent the i-th values of *x* and *y*, respectively, and $\bar{x}$ and $\bar{y}$ represent the mean values of *x* and *y*, respectively.

## 3 Result

### 3.1 Water phase changes in soaking maize kernels

**Figure 3:**
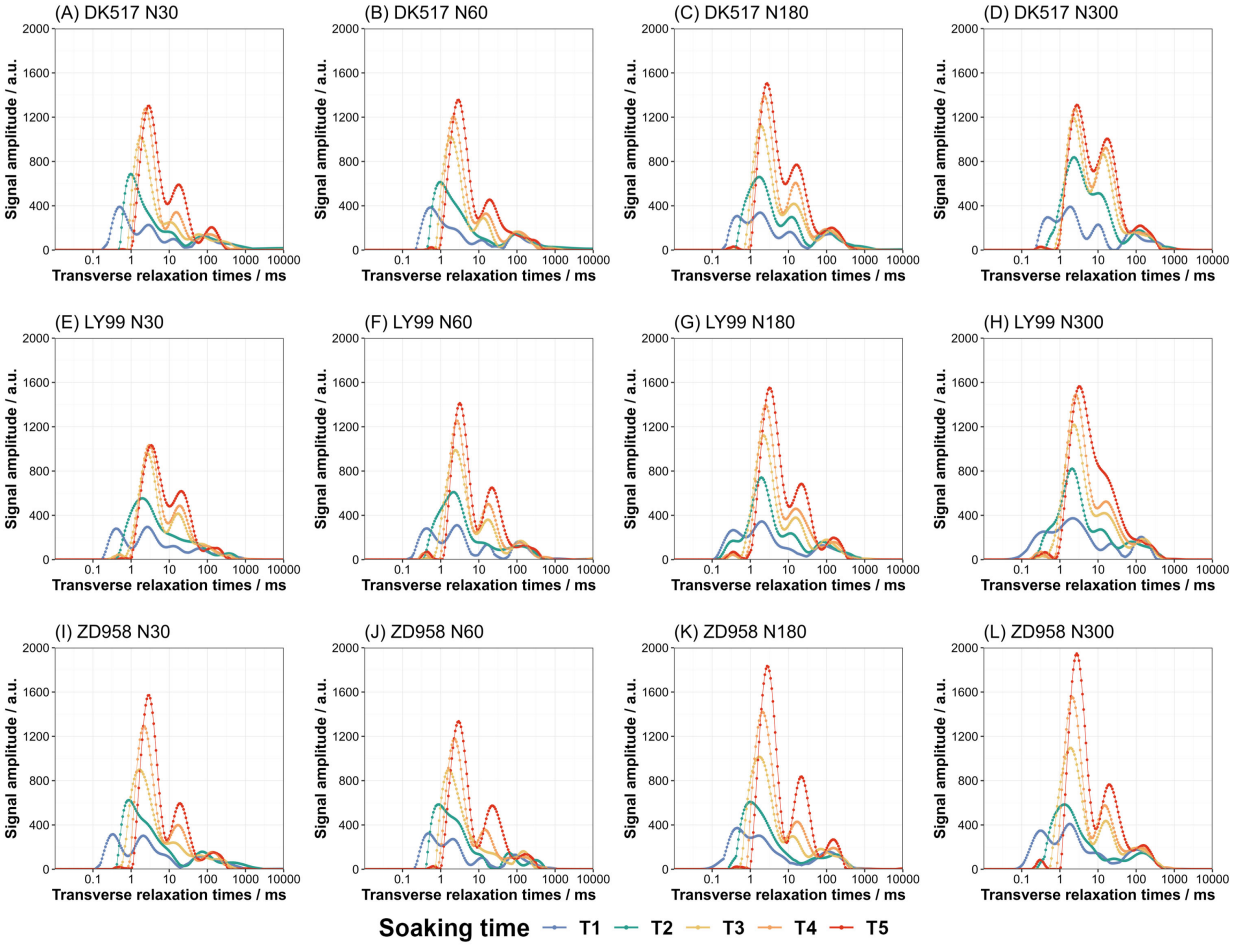
Dynamic changes in the LF-NMR relaxation spectrum of maize kernels under different nitrogen treatments during soaking. The lines of different colors represent the following soaking durations: T1 (blue line, 1 hour), T2 (green line, 3 hours), T3 (yellow line, 6 hours), T4 (orange line, 9 hours), and T5 (red line, 18 hours).The facet plots display the results of the T2 relaxation spectra for three maize cultivars: DK517 **(A–D)**, LY99 **(E–H)**, and ZD958 **(I–L)** under different nitrogen levels of N30, N60, N180, and N300.

The analysis of *T_2_* relaxation spectra at various soaking durations reveals the distribution and transition of water phase within the maize kernel. The LF-NMR *T*_2_ relaxation time distribution of maize kernels for different cultivars under different nitrogen treatments soaking with different durations are shown in **Figure 3**. The amplitude of signal shows typical pattern with several peaks in maize kernels (**Figure 3**). With soaking duration increased, these peaks evolved into three distinct categories: bound water, semi-bound water, and free water. Bound water exhibited the strongest signal intensity, followed by semi-bound water, with free water showing the weakest signal. As soaking time increases, the peaks shifted progressively to the right of the x-axis, indicating a transition in water phase, especially as the bound and semi-bound water signals intensify. Both the maximum amplitude of signal and the corresponding relaxation time increased with increasing soaking time across cultivars and nitrogen treatments. The maximum amplitude of signal increased with increasing nitrogen rate. Under low nitrogen rate, the maximum signal was 1328.40 a.u. at 2.97 ms on average for DK517, and 1221.89 a.u. at 3.30 ms for LY99, and 1453.01 a.u. at 2.97 ms for ZD958. Under high nitrogen rate, the maximum signal was 1407.56 a.u. at 2.77 ms on average for DK517, and 1557.60 a.u. at 3.18 ms for LY99, and 1890.64 a.u. at 2.77 ms for ZD958.

### 3.2 Proportions of water in different phases in soaking maize kernels

**Figure 4:**
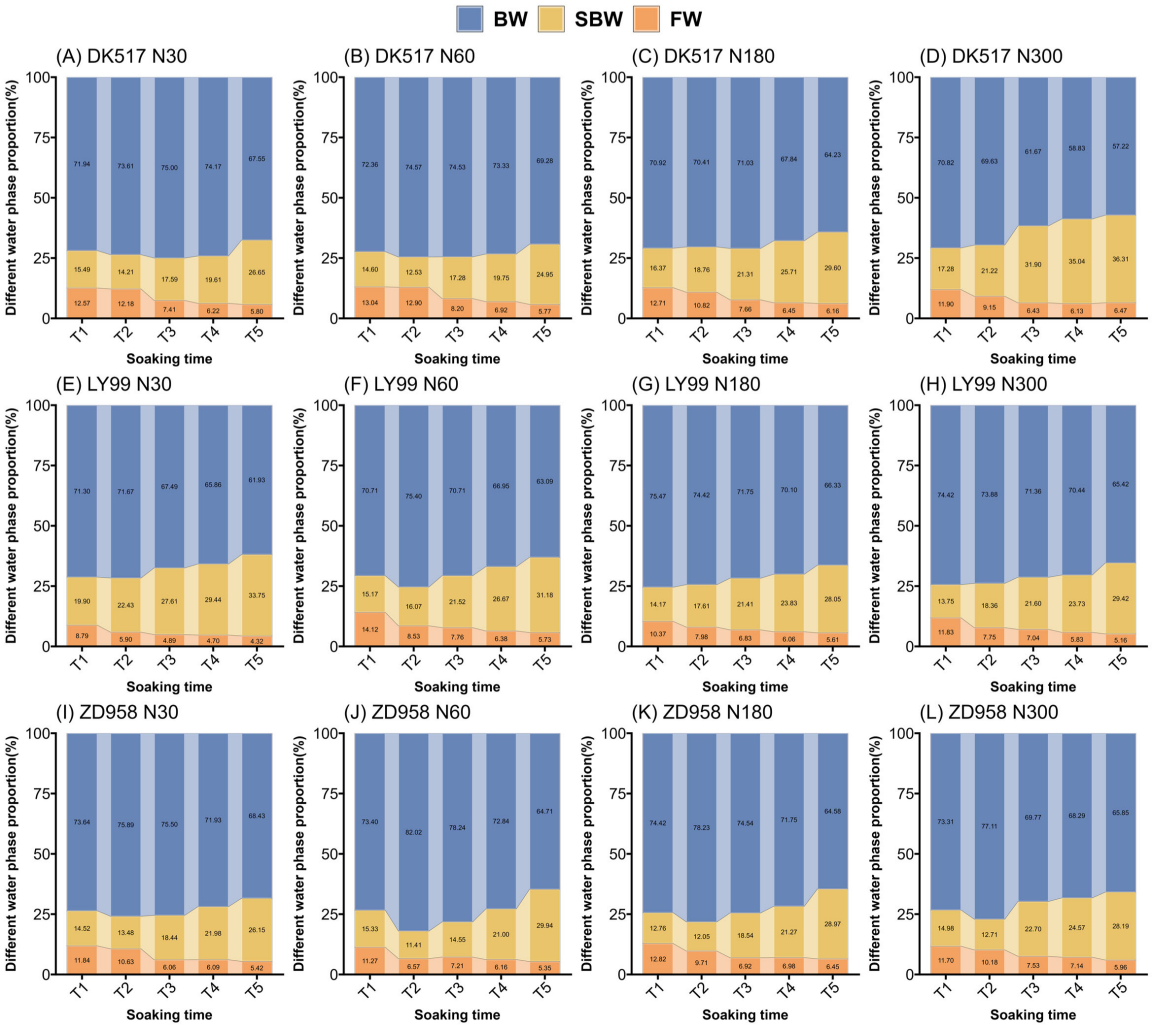
Dynamic changes in the proportion of water phases in maize kernels of DK517 **(A–D)**, LY99 **(E–H)**, and ZD958 **(I–L)** under different nitrogen treatments during soaking. Different colors in a bar represent the respective water phases: blue indicates bound water (BW), yellow represents semi-bound water (SBW), and orange denotes free water (FW).

The soaking time, cultivar, nitrogen rate significantly affected BWP, SBWP, and FWP (**Table 2**). Nitrogen rate significantly interacted with cultivars, indicating that the nitrogen effects on kernel water phase were influenced by cultivars. BWP generally increased initially, reaching a maximum in stages T1, T2, or T3, before gradually decreasing (**Figure 4**). At the T5 stage, the BWP of DK517 under different nitrogen treatments decreased by 9.93%, 7.10%, 9.57%, and 19.20%, respectively, compared to the maximum proportion at the time of submergence, with the greatest decrease at the nitrogen application rate of N300 (**Figures 4A–D**). LY99 showed reductions of 13.58%, 16.33%, 12.10%, and 12.10%, with the highest reduction at N60 (**Figures 4E–H**), while ZD958 decreased by 9.83%, 21.11%, 17.45%, and 14.60%, with the largest reduction at N60 (**Figures 4I–L**). Conversely, SBWP showed an initial decrease, reaching its lowest level during T1 and T2 before gradually increasing. At T5, the SBWP in DK517 increased by 87.53%, 99.12%, 80.85%, and 110.15% from its minimum, with the highest increase at N300 (**Figures 4A–D**). LY99 increased by 69.54%, 105.61%, 98.00%, and 114.01% (**Figures 4E–H**), while ZD958 saw increases of 93.98%, 162.38%, 140.32%, and 121.79%, with the largest gain at N60 (**Figures 4I–L**). The FWP was highest at T1, showing a steady decline over time. By T5, the FWP in DK517 had decreased by 53.88%, 55.75%, 51.50%, and 48.45% across nitrogen treatments, with the most pronounced reduction at N60 (**Figures 4A–D**). LY99’s FWP dropped by 50.86%, 59.42%, 45.85%, and 56.39%, with the greatest decline at N60 (**Figures 4E–H**), while ZD958’s FWP decreased by 54.27%, 52.53%, 49.66%, and 49.04%, with the largest drop at N30 (**Figures 4I–L**). These findings suggest that the predominant water phase in the kernel is bound water. However, with prolonged soaking durations, the proportion of bound and free water decreases, while the proportion of semi-bound water increases.

**Table 2:** Results of ANOVA analysis of soaking time, cultivars, nitrogen application rate, and their interactions on internal moisture and structural traits of maize kernel.

Traits’ category	Assessment index	Variance						
		ST	C	N	ST×C	ST×N	C×N	ST×C×N
Water phase in kernel	MC	**	**	ns	**	ns	ns	ns
	BWP	**	**	**	ns	ns	**	ns
	SBWP	**	**	**	ns	ns	**	ns
	FWP	**	**	**	**	ns	**	ns
	TWC	**	ns	**	ns	**	ns	ns
	BWC	**	ns	**	ns	**	ns	ns
	SBWC	**	ns	**	ns	*	*	ns
	FWC	**	*	**	*	**	*	ns
Internal structure in kernel	KV		**	**			*	
	FEV		*	ns			*	
	VEV		ns	**			ns	
	ENDV		*	**			ns	
	EMBV		*	**			*	
	FEP		ns	ns			*	
	VEP		*	ns			ns	
	ENDP		ns	ns			ns	
	EMBP		ns	ns			ns	

**P< 0.01, *P< 0.05, and ns ≥ 0.05.

Maize kernel MC were significantly affected by soaking time (ST), cultivars (C), and their interaction (ST×C) (P<0.01) (**Table 2**). For DK517 kernels, the average moisture content at successive soaking times was 7.77%, 16.11%, 22.04%, 25.11%, and 29.04%. In LY99 kernels, these values were 8.78%, 17.02%, 23.38%, 26.69%, and 31.03%, respectively, while ZD958 kernels showed mean moisture contents of 6.78%, 13.14%, 18.94%, 23.04%, and 28.43% at each soaking stage (**Supplementary Figure S1**). BWP for DK517 was significantly decreased by increasing nitrogen rate with a minimal of 61.67%, 58.83%, and 57.22% under N300 in T3, T4, and T5 (**Supplementary Table S1**). Varietal differences in BWP were primarily significant at T2, with the descending order ZD958 > LY99 > DK517. SBWP for ZD958 was significantly increased by increasing nitrogen rate with a maximal of 21.22%, 31.90%, 35.04%, and 36.31% under N300 in T2, T3, T4, and T5. BWP and SBWP under different nitrogen rate for LY99 and ZD958 was not significantly differed except for T3 for ZD958. LY99 had a significantly higher SBWP than other cultivars under N30 in most soaking durations. FWP was significantly increased by increasing nitrogen rate for LY99 but not affected for DK517 and ZD958.

### 3.3 Water content in different phases in soaking maize kernels

The soaking time and nitrogen rate significantly affected TWC, BWC, SBWC, and FWC (**Table 2**). Soaking time significantly interacted with nitrogen rate, indicating that the nitrogen effect on water content in different phases were influenced by soaking time. The water content of maize kernels generally increased with prolonged soaking (**Table 3**). TWC was significantly increased by increasing nitrogen rate across cultivars (**Table 3**). BWC was significantly increased by increasing nitrogen rate across soaking durations and cultivars. BWC reaches the maximum value of 90.32 mg, 109.71 mg, and 107.34 mg under N180, N300, and N300 at T5 for DK517, LY99, and ZD958 respectively. SBWC under N300 was significantly higher than that under low nitrogen rate (N30 and N60 at T2, N30 at T3, N30, N60 and N180 at T4, N60 at T5) for DK517, but not differed between nitrogen rate for LY99 and ZD928, indicating the significant interaction between cultivars and nitrogen (*P*<0.05, **Table 3**). Compared to TWC, FWC showed a similar pattern of response to nitrogen across cultivars, with peaking at N300 with values of 9.92 mg, 8.65 mg, and 9.53 mg for DK517, LY99, and ZD958, respectively.

**Table 3:** Comparison of mean values of water content and water phase content in maize kernels between cultivars and nitrogen treatments under different soaking time.

ST	N	TWC (mg)			BWC (mg)			SBWC (mg)			FWC (mg)		
		DK517	LY99	ZD958	DK517	LY99	ZD958	DK517	LY99	ZD958	DK517	LY99	ZD958
T1	N30	21.1Aa	28.33Aa	25.33Aa	14.77Aa	19.94ABa	18.65Aa	3.52Aa	5.85Aa	3.62Aa	2.71Aa	2.54Aa	3.06Aa
	N60	24.6Aa	24.33Aa	22.67Aa	17.61Aa	17.11Ba	16.68Aa	3.55Aa	3.73Aa	3.46Aa	3.18Aa	3.50Aa	2.52Aa
	N180	29.33Aa	26.67Aa	26.00Aa	20.77Aa	19.97ABa	19.36Aa	4.83Aa	3.89Aa	3.32Aa	3.73Aa	2.81Aa	3.32Aa
	N300	31.67Aa	35.67Aa	23.33Aa	22.71Aa	26.46Aa	17.09Aa	5.60Aa	5.00Aa	3.51Aa	3.68Aa	4.21Aa	2.73Aa
T2	N30	47.10Ba	58.33Aa	51.33Aa	34.23Ba	40.84Aa	38.95Aa	7.04Ba	14.07Aa	6.92Aa	5.73Aa	3.42Bb	5.46Aa
	N60	48.6Ba	52.00Aa	46.33Aa	35.94Ba	39.07Aa	38.00Aa	6.08Ba	8.46Aa	5.30Aa	6.31Aa	4.48ABab	3.04Bb
	N180	60.33Ba	60.67Aa	50.00Aa	42.09Ba	44.98Aa	39.24Aa	11.38Ba	10.83Aa	5.98Aa	6.52Aa	4.86ABa	4.78ABa
	N300	90.00Aa	73.33Aa	53.33Aa	61.91Aa	54.12Aab	41.10Ab	20.52Aa	13.52Aab	6.81Ab	7.56Aa	5.70Aab	5.43Ab
T3	N30	69.77Ba	82.67Aa	73.67ABa	51.58Ba	54.97Ba	55.68ABa	12.90Ba	23.72Aa	13.51Aa	5.18Ba	3.97Ba	4.48Ba
	N60	68.60Ba	82.00Aa	65.33Ba	51.17Ba	57.98Ba	51.08Ba	11.88Ba	17.66Aa	9.51Aa	5.62ABa	6.36Aa	4.74Ba
	N180	96.67ABa	91.33Aa	79.33ABa	67.77ABa	65.39ABa	59.13ABa	21.53Ba	19.67Aa	14.71Aa	7.36Aa	6.27Aa	5.50Ba
	N300	126.00Aa	107.33Aa	100.67Aa	76.28Aa	76.61Aa	70.00Aa	42.15Aa	23.15Ab	23.11Ab	7.57Aa	7.57Aa	7.56Aa
T4	N30	86.10Ba	94.33Ba	93.67ABa	63.13BCa	63.30Ba	67.28ABa	17.77Aa	24.97Aa	19.85Aa	5.43BCa	6.07Aa	6.54ABa
	N60	82.60Ba	96.33ABa	83.33Ba	54.61Ca	70.19ABa	49.27Ba	24.50Aa	20.22Aa	28.89Aa	3.89Ca	5.92Aa	5.17Ba
	N180	115.33ABa	114.00ABa	106.67ABa	82.68ABa	77.05ABa	75.22Aa	25.57Aa	29.58Aa	25.25Aa	7.08Ba	7.37Aa	6.2ABa
	N300	142.67Aa	130.00Aa	125.00Aa	104.48Aa	91.12Aa	85.35Aa	28.40Aa	30.99Aa	30.80Aa	9.79Aa	7.89Aa	8.85Aa
T5	N30	108.77Ba	110.33Ca	120.33BCa	71.96Ba	66.98Ba	82.35BCa	30.63Ba	38.73Aa	31.47Aa	6.41Ba	4.62Ba	6.51Ba
	N60	107.27Ba	118.00BCa	111.67Ca	74.35ABa	74.46Ba	72.29Ca	26.78Ba	36.77Aa	33.42Aa	6.20Ba	6.77Aa	5.96Ba
	N180	142.67Aa	144.67ABa	147.33ABa	90.32Aa	95.75Aa	95.18ABa	43.54ABa	40.82Aa	42.67Aa	8.81Aa	8.10Aa	9.49Aa
	N300	156.33Aa	167.67Aa	162.33Aa	88.66ABb	109.71Aa	107.34Aa	57.76Aa	49.31Aa	45.47Aa	9.92Aa	8.65Aa	9.53Aa

At each water absorption time point, uppercase letters indicate significant differences (*P*< 0.05) between different nitrogen treatments within the same Cultivars, while lowercase letters indicate significant differences (*P*< 0.05) between cultivars under different nitrogen application levels, based on Duncan’s Significant Studentized Range (SSR) test.

TWC represents for water content, BWC for bound water content, SBWC for semi-bound water content, FWC for free water content.

### 3.4 Internal structure and its correlation to water phase distribution in maize kernels

The KV and EMBP were found to be significantly affected by cultivars, nitrogen rate and their interaction (**Table 2**). FEV was significantly influenced by cultivars and its interaction with nitrogen rate. VEV was significantly influenced by nitrogen rate. ENDV was significantly affected by both cultivars and nitrogen rate. KV under high nitrogen rate (811.97 mm^3^, 798.37 mm^3^, 857.43mm^3^) was significantly greater than that under low nitrogen rate (677.38 mm^3^, 557.29 mm^3^, 636.07 mm^3^) for DK517, LY99, and ZD958 (**Table 4**). ENDV and EMBV showed similar patterns with values of 597.18 mm^3^, 80.20 mm^3^ for DK517, 501.16 mm^3^, 56.12 mm^3^ for LY99, and 565.59 mm^3^, 70.48 mm^3^ for ZD958 under low nitrogen rate, and that of 723.46 mm^3^, 88.51 mm^3^ for DK517, 711.89 mm^3^, 86.48 mm^3^ for LY99, and 752.60 mm^3^, 104.82 mm^3^ for ZD958 under high nitrogen rate. FEV was significantly increased by increasing nitrogen rate for DK517 and LY99, but not for ZD958. VEV was significantly increased by increasing nitrogen rate for LY99 and ZD958, but not for DK517. Only VEP was significantly affected by cultivars, while other proportions were not significantly affected by neither the cultivars nor nitrogen rate, suggesting a stable spatial distribution inside maize kernel.

**Table 4:** Structural traits of internal structure of maize kernel based on μ-CT scanning technology.

Cultivars	N	Kernel phenotyping indices				
		FEV (mm^3^)	VEV (mm^3^)	ENDV (mm^3^)	EMBV (mm^3^)	KV (mm^3^)
DK517	N30	147.22Bab	452.15Aa	599.38Ba	87.94Aa	687.31Ba
	N60	156.97Ba	438.00Aa	594.97Ba	72.47Aa	667.44Ba
	N180	237.74Aa	497.79Aa	735.52Aa	92.00Aab	827.52Aa
	N300	162.68Ba	548.73Aa	711.41Aa	85.01Ab	796.42Ab
LY99	N30	80.34Bb	403.69Ba	484.03Cb	47.61Cb	531.64Cb
	N60	79.44Bb	438.86Ba	518.30Ca	64.64BCa	582.94Ca
	N180	161.54Ab	501.33ABa	662.87Ba	72.96Bb	735.83Ba
	N300	169.55Aa	591.37A	760.92Aa	100.00Aab	860.92Aab
ZD958	N30	153.06Aa	439.44Ba	592.50Ba	75.57Ba	668.07Ba
	N60	187.16Aa	351.53Ba	538.69Ba	65.38Ba	604.07Ba
	N180	140.76Ab	579.81Aa	720.58Aa	97.96Aa	818.54Aa
	N300	125.58Aa	659.06Aa	784.63Aa	111.68Aa	896.32Aa

In the same column, uppercase letters indicate significant differences (P< 0.05) between different nitrogen treatments within the same cultivars, while lowercase letters indicate significant differences (P< 0.05) between cultivars under different nitrogen application levels, based on Duncan’s Significant Studentized Range (SSR) test.

**Figure 5:**
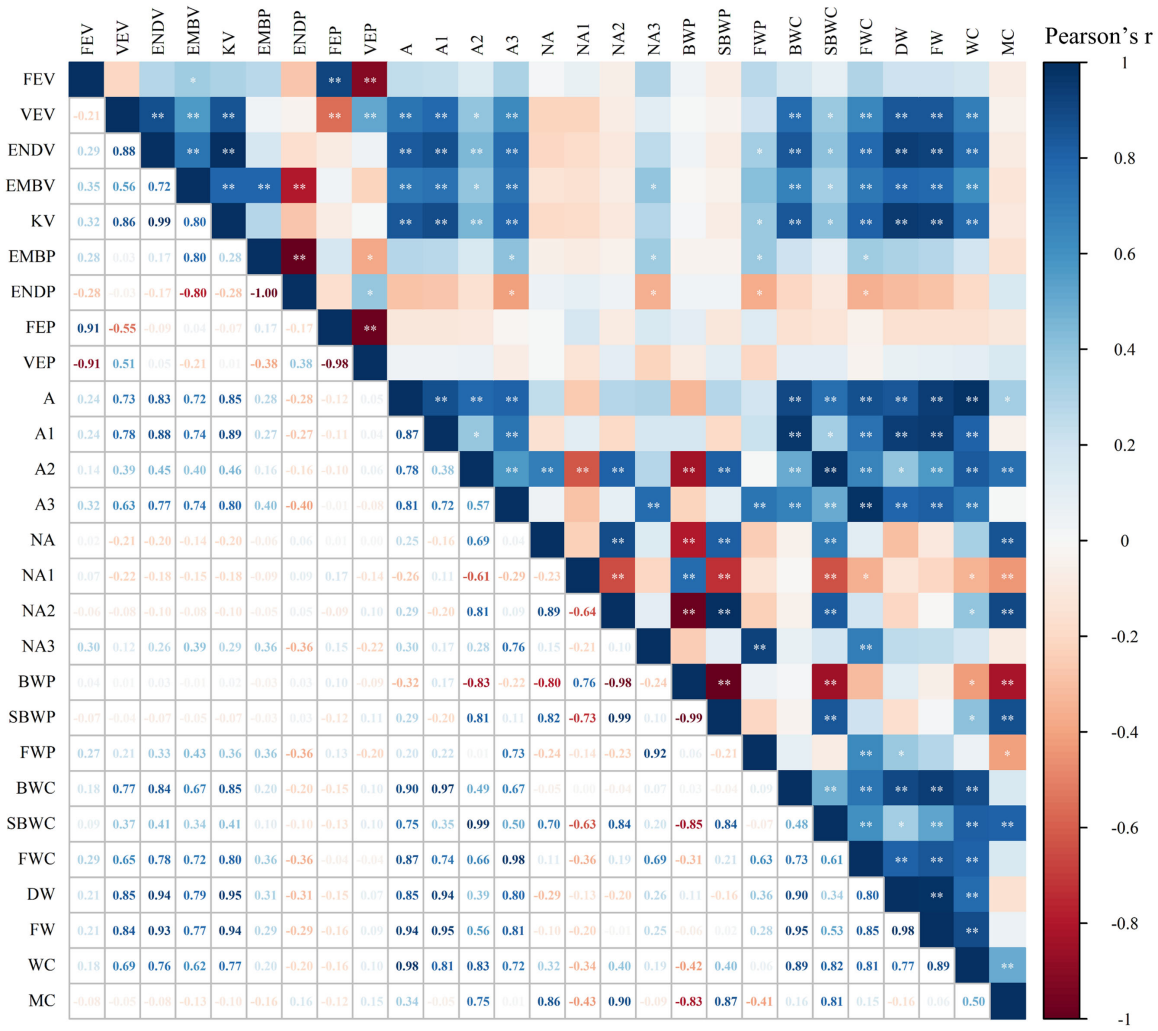
Correlation heatmap of maize kernel internal spatial structure and water phase at the T5 stage. At the T5 stage, maize grains are fully soaked and gradually reach saturation, with a stable water phase distribution. P<0.01, ** highly significant correlation, P<0.05, * significant correlation. In addition, NA, NA1, NA2, and NA3 represent the total area, bound water area, semi-bound water area, and free water area normalized by the fresh weight of the individual kernel.

**Figure 6:**
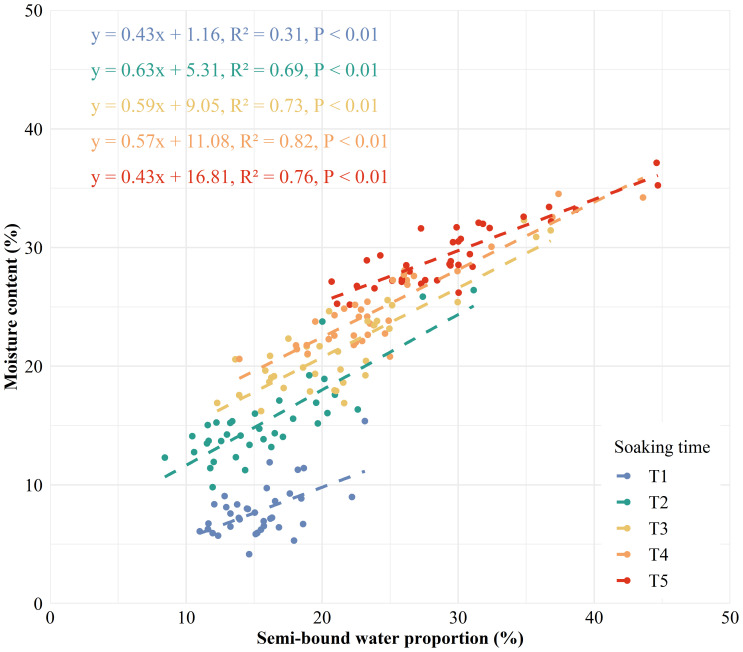
Linear relationship between the proportion of semi-bound water and moisture content in maize kernels at different soaking times (blue for T1, green for T2, yellow for T3, orange for T4, and red for T5). *P<* 0.01 indicates a highly significant relationship.

A strong correlation was found between water phase distribution and the internal structure of kernels (**Figure 5**). A significant positive correlation was observed between A and WC, and between NA and MC in maize kernels (**Supplementary Figure S2**), demonstrating the efficacy of LF-NMR in measuring water content of individual kernel. BWC, SBWC, and FWC showed significantly positive correlations to VEV, ENDV, EMBV, and KV, suggesting a strong relationship between water content in difference phase and internal structure of maize kernel. BWC and SBWC correlates most strongly with KV (0.85, 0.41), followed by ENDV (0.84, 0.41), VEV (0.77, 0.37), and EMBV (0.67, 0.34). However, FWC correlates most strongly with KV (0.80), followed by ENDV (0.78), EMBV (0.72), and VEV (0.65). MC was found to correlate most strongly with NA2 (0.90), followed by SBWP (0.87), NA (0.86), SBWC (0.81), and A2 (0.75), suggesting that signal of semi-bound water might be a robust indicator to predict moisture content in maize kernel. SBWP showed a linear relationship with MC across soaking time, but the relationship was stronger for higher MC levels (R^2^ = 0.69-0.82 for MC > 10%) than lower MC levels (R^2^ = 0.31 for MC< 10%) (**Figure 6**). The significantly negative correlations between BWP and SBWP (-0.99), SBWC (-0.85), and MC (-0.83) demonstrate a clear trend of increasing semi-bound water proportion with elevated water content (**Figure 4**).

### 3.5 Water signal distribution imaging and μ-CT scanning of different maize kernels

**Figure 7:**
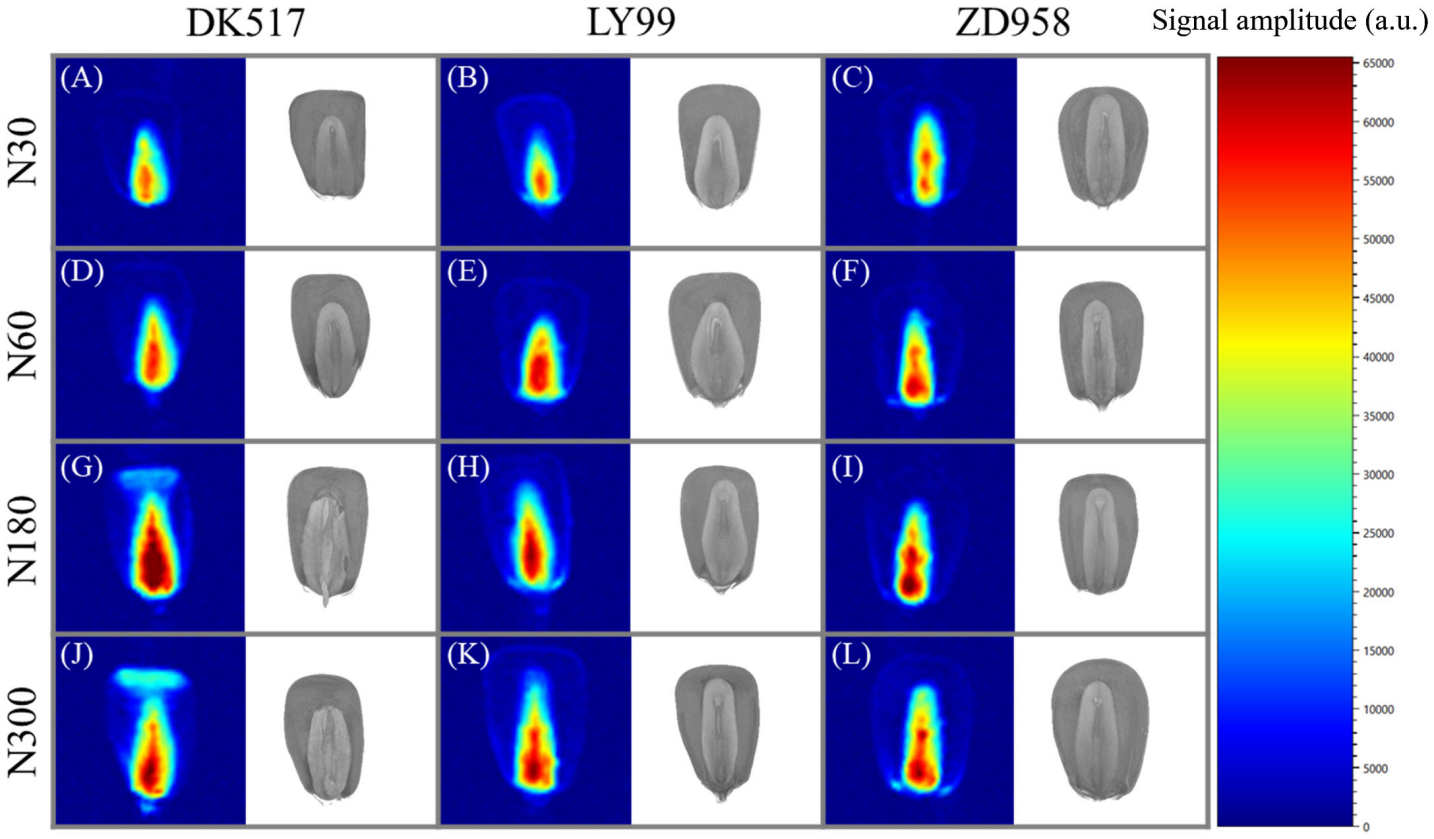
Visualization of moisture distribution and structural morphology in maize kernels after 18 hours of soaking based on LF-NMR and μ-CT scanning. **(A–C)** represent the moisture distribution at N30, **(D–F)** at N60, **(G–I)** at N180, and **(J–L)** at N300. The pseudocolor images represent the MRI scans of the kernels, while the grayscale images depict the μ-CT scans. The legend indicates the signal values corresponding to moisture content within the kernels.

Visual comparison between water distribution and internal structure in individual kernel for different cultivars under different nitrogen rate were shown in **Figure 7**. The *T_2_* signal in the embryo area was substantially stronger than other tissues, showing a gradually diminishing pattern from the base towards surrounding tissues across all cultivars and nitrogen treatments. This indicates that water may infiltrate the kernel via embryo and then gradually distribute into floury endosperm. The signal within the embryo region increased substantially with increasing nitrogen application rate across all cultivars. DK517 exhibited a significantly higher signal at the kernel top under high nitrogen, suggesting a different water distribution pattern outside the embryo compared to LY99 and ZD958.

## 4 Discussion

The development of yield and quality in kernel crops involve the accumulation of substances such as starch, proteins, and fats, which interact with water within the kernel. Given the spatial distribution of these substances and the varied roles of water phases, we hypothesized that water phase distribution is influenced by kernel internal structure. Maize kernels with varying water phase content and internal structure were generated by soaking kernels for different cultivars at various nitrogen rates for different durations. We characterized the *T*_2_ relaxation time distribution pattern of maize kernel and its response to soaking time, cultivars, and nitrogen application rate by LF-NMR. Further integration to μ-CT revealed that fraction of different water phase and volume of different tissues in kernel were found significantly affected by cultivar and nitrogen rate. A strong relationship between water content in difference phase and volume of different tissues in maize kernel was quantified. We also found that indicators related to semi-bound water might serves as robust predictors of moisture content in maize kernel.

### 4.1 Water phase characteristics of maize kernels

The LF-NMR technique is a method based on the spin properties of the hydrogen nucleus in a magnetic field, which makes it possible to jump from a high energy state to a low energy state in a non-radiative way. By measuring the absorption of the resonant radio frequency by the nuclear spin of the proton, LF-NMR can monitor proton states with different relaxation times and their distribution (Carneiro et al., 2013). Because hydrogen protons are abundant in water molecules, LF-NMR analysis of relaxation time changes allows for a quantitative and qualitative study of water content, distribution, and migration within materials (Kamal et al., 2019). Meanwhile, there was a good correlation between the water content of the samples during the soaking and the amplitude of the *T_2_* inversion spectrum signal in LF-NMR (**Supplementary Figure S2**), affirming the reliability of LF-NMR for examining maize kernel water status. Notably, signal peaks of each water phase shifted rightward with longer soaking times, and peak intensities increased (**Figure 3**), suggesting patterns of water migration similar to those found in other crops. Studies on rice have demonstrated similar rightward shifts in both bound and free water peaks with prolonged soaking (Wang et al., 2024), suggesting that water dynamics during absorption may be consistent across different crops. Additionally, the position of the *T_2_* relaxation inversion peak in LF-NMR reflects the kinetic properties of kernel moisture, particularly the degree of binding between water molecules and macromolecules (Mou et al., 2016).

### 4.2 Water uptake and distribution in soaking maize kernels

The soaking process of maize kernels conforms to Fick’s law of diffusion, where the diffusion rate gradually decreases over time until equilibrium moisture content is reached (Antwi et al., 2022). Studies have shown that temperature significantly affects water absorption rates (Nweke, 2010). However, this study excluded temperature as a variable and focused on the impact of kernel structure on water absorption behavior in different maize cultivars. This study found that the initial water absorption rates of different maize cultivars (e.g., DK517, LY99, and ZD958) were 2.88%.h^-1^, 4.12%.h^-1^, and 3.18%.h^-1^, respectively, during the first 3 hours (**Supplementary Figure S1**). McDonald et al. (1994) reported that kernels exhibit rapid water uptake within the first 6 hours, and our findings are consistent with this result. Additionally, the high-protein maize cultivar LY99 exhibited higher kernel moisture content compared to other cultivars (**Table 1**; **Supplementary Figure S1**). This is likely because smaller kernels generally have a larger surface area-to-volume ratio, enabling faster water absorption, while kernels with higher protein or starch content exhibit stronger water-binding capacities (Schopf and Scherf, 2021; Tarahi et al., 2022). This highlights the critical role of water absorption pathways and internal structure in maize kernels.

The mechanism of water uptake by maize kernels during soaking is a complex process involving both physical and chemical changes. It is widely accepted that water initially penetrates the pericarp, then moves into the endosperm, and eventually diffuses into the embryo. Agarry et al.(2014) reported that water uptake primarily occurs through capillary action in the outer pericarp, resulting in a rapid increase in kernel moisture. Our LF-NMR visualization further confirmed this, showing strong signals in the embryo region (**Figure 7**), which aligns with the findings of Chen et al. (2020). Similarly, Miano et al. (2017) suggested that moisture first reaches the embryo and then spreads to the endosperm during early stages of soaking. Additionally, Meyer et al. (2007) and Penfield (2017) observed similar water absorption pathways in soybean and rapeseed. Thus, LF-NMR proves to be an effective tool for tracking water migration within kernels. Additionally, we observed a positive correlation between kernel volume and water content, likely due to larger seeds having a greater surface area for water absorption. The significant positive correlation between ENDV and WC in this study also suggests that the endosperm, as a major storage site for macromolecules like starch and proteins, plays a crucial role in binding water and enhancing water uptake. This finding is consistent with previous studies (Uriarte-Aceves and Sopade, 2023).

The process of water soaking in maize kernels is mainly driven by osmotic pressure differences, where the osmotic pressure of the external water is lower than that inside the kernel, prompting the movement of water towards the interior of the kernel. In the present study, it was found that kernel moisture increased with duration of soaking until osmotic pressure stabilized (**Supplementary Figure S1**). The loose structure of the endosperm facilitates the rapid entry of water into the cell (Wen et al., 2017), providing a physical basis for water uptake and distribution. This is supported by the fact that the internal structural properties of the kernel were significantly correlated with water phase distribution (**Figure 5**). Bound and semi-bound water are easily bound to macromolecules and are usually found in the nutrient-rich regions of the kernels (Nuss and Tanumihardjo, 2010; Xu et al., 2019b; Wang et al., 2019). The correlation between bound water and horny endosperm was higher than that of powdered endosperm, probably because the protein content and spatial distribution were related to horny endosperm (Xu et al., 2019b), reflecting the interaction between water and nutrients. During the soaking process, the proportion of bound water gradually decreased, and the proportion of semi-bound water increased, indicating that water began to enter the cell interior. During this process, the water entering the kernel interacts with macromolecules. Once the bound water has fully interacted with the macromolecules, the additional water entering the kernel exists in a state between free water and bound water, leading to an increase in the proportion of semi-bound water. The small increase in the proportion of free water may be due to the entry of water into the voids and cracks of the kernels through capillary action. The moisture signals of the embryo were stronger than those of the chalky endosperm and horny endosperm, which was further confirmed by the significant correlation between the horny endosperm and the moisture of the kernels (**Figures 5**, **7**). In conclusion, by exploring the distribution characteristics of kernels structure and water phase, we can reveal the migration characteristics of moisture in the kernels and provide new perspectives for the study of grain quality.

### 4.3 Kernel moisture as a function of semi-bound water

Our results are consistent with the existing literature, particularly with regard to the effect of nitrogen application on water status. The BWP increased with higher nitrogen applications in the study by Krishnan et al. (2014). However, we observed that the ratio of semi-bound to free water was not fully consistent with the previous study with increasing nitrogen application. This may be related to present study of the soaking process of the dry kernels in reverse order, highlighting the differences that can occur under different experimental conditions. For instance, Chen et al. (2020) examined kernel dehydration, water uptake, and rehydration, focusing on dehydration, whereas this study’s focus on soaking highlighted differences in water dynamics. By comparing the water uptake in kernels across different maize cultivars, this study aims to elucidate the response characteristics of water phase in kernels to internal structure and nutrient elements. Overall, these detailed observations on maize kernel water phase dynamics provide insights into water content variability under nitrogen treatments, offering a foundation for similar investigations in other crops.

### 4.4 Impact of nitrogen application on water phase distribution in maize kernels

Nitrogen treatments significantly impact the content of various water phases in maize kernels (**Table 2**). Mou et al. (2016) found that bound water content indirectly reflects dry matter content in wheat, as water phase distribution is closely tied to soluble sugar and starch content. Similar connections between kernel water phases and nutritional components have been observed in maize (Duan et al., 2024). In this study, absolute water content in each phase is influenced by kernel moisture and macromolecule-water binding strength. Increased nitrogen application leads to higher nitrogen accumulation, lipid, and protein contents in kernels (Hammad et al., 2023; Feng et al., 2024). These findings suggest that nitrogen-induced differences in water phase content stem from changes in kernel internal composition. The higher absolute content of bound water under high nitrogen conditions (**Table 3**) aligns with findings by Krishnan et al. (2014). This study validates the reliability of using water phase distribution analysis to explore internal kernel components and demonstrates the potential of LF-NMR in evaluating maize kernel quality.

### 4.5 Limitations and future directions

Our study focuses on water phase transitions and their interactions with kernel structures during soaking experiments. While the soaking process differs from natural dehydration, the findings provide valuable insights into water dynamics within kernels. For example, the transformation of bound water to semi-bound water observed in this study reflects the interaction between water molecules and macromolecules, which may also occur during natural dehydration as kernels lose moisture through evaporation and internal transport systems (Zhang et al., 2022). To our knowledge, this study is the first attempt to employ LF-NMR and μ-CT techniques to investigate moisture phase distribution in maize kernels and confirm the feasibility of exploring the interactions between water dynamics and internal structure in maize kernels. However, several limitations exist and need to be addressed in future research.

First, the internal structural dependence of water phase in maize kernel was drawn from soaking kernels for various duration, but whether it is still held for natural dehydration process of maize kernel remain unknown. Future attention should be paid on the relationship between water phase distribution and internal morphological structures during kernel filling. Second, the distribution of different water phases was inferred from the amplitude of correlation coefficient and the spatial visualization of *T_2_* signal of total water within a kernel (**Figure 7**). The distribution of water phases and metabolic substances needs to be investigated in detail by 3D-MRI and hyperspectral microscope imaging techniques (Zhang et al., 2024). At last, kernel moisture changes are often quantified through empirical modeling with limited underlying mechanisms (Chazarreta et al., 2023). Computational biology models, such as metabolic transport and water diffusion model, should be integrated to gain a comprehensive understanding of kernel development.

## 5 Conclusion

This study combined LF-NMR and μ-CT to investigate the relationship between water phase distribution and internal structure in soaking maize kernels. Our findings reveal that water in maize kernel majorly stored in bound water, and its proportion decreases with prolonged soaking duration. Based on the quantitative relationship between water content in different phases and the tissue volume within maize kernel, we identified potential water movement pathways during soaking, primarily through the pedicel into the embryo and secondarily through the pericarp into the vitreous endosperm. Notably, the proportion of semi-bound may water serves as a reliable indicator for predicting kernel moisture content, offering a new method for rapid assessment on kernel moisture. The study provides a preliminary understanding of the structural basis of water distribution in maize kernels, thereby opening up the potential for designing efficient production systems and breeding cultivars well-suited for mechanical harvesting.

## Data Availability

The raw data supporting the conclusions of this article will be made available by the authors, without undue reservation.
