# Supplementary material for: Water phase distribution and its dependence on internal structure in soaking maize kernels: a study using low-field nuclear magnetic resonance and X-ray micro-computed tomography
Source: Front Plant Sci. 2025 Jan 24;15:1529514. doi: 10.3389/fpls.2024.1529514 (PMC11802422; doi:10.3389/fpls.2024.1529514)
Supplement: Supplementary file 1 [file Supplementaryfile1.docx]

**Supplementary Table 1.** Comparison of mean values of moisture content and water phase proportions in maize kernels between cultivars and nitrogen treatments under different soaking time. MC represents for moisture content, BWP for bound water proportion, SBWP for semi-bound water proportion, FWP for free water proportion.

| ST | N | MC (%) | | | BWP (%) | | | SBWP (%) | | | FWP (%) | | |
| --- | --- | --- | --- | --- | --- | --- | --- | --- | --- | --- | --- | --- | --- |
|  |  | DK517 | LY99 | ZD958 | DK517 | LY99 | ZD958 | DK517 | LY99 | ZD958 | DK517 | LY99 | ZD958 |
| T1 | N30 | 6.84Ab | 10.62Aa | 7.53Aab | 71.94 Aa | 71.30 Aa | 71.30 Aa | 15.49Ab | 19.90Aa | 14.52Ab | 12.57Aa | 8.79Cb | 11.84Aa |
|  | N60 | 7.70Aa | 8.44Aa | 7.84Aa | 72.36 Aa | 70.71 Aa | 70.71 Aa | 14.60Aa | 15.17ABa | 15.33Aa | 13.04Aa | 14.12Aa | 11.27Aa |
|  | N180 | 8.00Aa | 7.51Aa | 6.44Aa | 70.92 Aa | 75.47 Aa | 75.47 Aa | 16.37Aa | 14.17ABa | 12.76Aa | 12.71Aa | 10.37BCa | 12.82Aa |
|  | N300 | 8.54Aa | 8.56Aa | 5.32Aa | 70.82 Aa | 74.42 Aa | 74.42 Aa | 17.28Aa | 13.75ABa | 14.98Aa | 11.90Aa | 11.83ABa | 11.70Aa |
| T2 | N30 | 14.16Bb | 19.91Aa | 14.60Ab | 73.61Aa | 71.67Aa | 75.89Aa | 14.21Bb | 22.43Aa | 13.48Ab | 12.18Aa | 5.90Ba | 10.63Aa |
|  | N60 | 14.22Aa | 16.42Aa | 14.81Aa | 74.57Ab | 75.40Aab | 82.02Aa | 12.53Ba | 16.07Aa | 11.41Aa | 12.90Aa | 8.53ABa | 6.57ABb |
|  | N180 | 15.16Aa | 15.49Aa | 11.64Aa | 70.41Ab | 74.42Aab | 78.23Aa | 18.76ABa | 17.61Aa | 12.05Aa | 10.82Aab | 7.98Ba | 9.71ABa |
|  | N300 | 20.92Aa | 16.26Aab | 11.51Ab | 69.63Ab | 73.88Aab | 77.11Aa | 21.22Aa | 18.63Aab | 12.71Ab | 9.15Ab | 7.75Aa | 10.18Aa |
| T3 | N30 | 19.66Bb | 26.06Aa | 19.17Ab | 75.00Aa | 67.49Ab | 75.50ABa | 17.59Bb | 27.61Aa | 18.44ABb | 7.41ABa | 4.89Bb | 6.06Aab |
|  | N60 | 19.00Ba | 23.59Aa | 19.68Aa | 74.53Aab | 70.71Ab | 78.23Aa | 17.28Aa | 21.52Aa | 14.55Ba | 8.20Aa | 7.76Aa | 7.21Aa |
|  | N180 | 22.30ABa | 21.75Aa | 17.34Aa | 71.03Aa | 71.75Aa | 74.54ABa | 21.31Aa | 21.41Aa | 18.54ABa | 7.66ABa | 6.83Aa | 6.92Aa |
|  | N300 | 27.20Aa | 22.14Aab | 19.58Ab | 61.67Bb | 71.36Aa | 69.77Ba | 31.90Aa | 21.60Ab | 22.70Ab | 6.43Ba | 7.04Aa | 7.53Aa |
| T4 | N30 | 23.14Bb | 28.79Aa | 23.17Ab | 74.17Aa | 65.86Ab | 71.93Aab | 19.61Bb | 29.44Aa | 21.98Aab | 6.22Aa | 4.70Ba | 6.09Aa |
|  | N60 | 22.09Ba | 26.64Aa | 23.82Aa | 73.33Aa | 66.95Aa | 72.84Aa | 19.75Ba | 26.67Aa | 21.00Aa | 6.92Aa | 6.38Aa | 6.16Aa |
|  | N180 | 25.38ABa | 25.72Aa | 21.96Aa | 67. 84Aa | 70.10Aa | 71.75Aa | 25.71Ba | 23.83Aa | 21.27Aa | 6.45Aa | 6.06ABa | 6.98Aa |
|  | N300 | 29.85Aa | 25.63Aab | 23.19Ab | 58.83Bb | 70.44Aa | 68.29Aa | 35.04Aa | 23.73Ab | 24.57Ab | 6.13Aa | 5.83ABa | 7.14Aa |
| T5 | N30 | 27.61ABa | 32.12Aa | 27.94Aa | 67.55Aa | 61.93Aa | 68.43Aa | 26.65ABa | 33.75Aa | 26.15Aa | 5.80Aa | 4.32Bb | 5.42Aab |
|  | N60 | 26.86Ba | 30.75Aa | 29.50Aa | 69.28Aa | 63.09Aa | 64.71Aa | 24.95Ba | 31.18Aa | 29.94Aa | 5.77Aa | 5.73Aa | 5.35Aa |
|  | N180 | 29.71ABa | 30.54Aa | 28.02Aa | 64.23ABa | 66.33Aa | 64.58Aa | 29.60ABa | 28.05Aa | 28.97Aa | 6.16Aa | 5.61ABa | 6.45Aa |
|  | N300 | 31.99Aa | 30.72Aa | 28.25Aa | 57.22Ba | 65.42Aa | 65.85Aa | 36.31Aa | 29.42Aa | 28.19Aa | 6.47Aa | 5.16ABb | 5.96Aab |

At each water absorption time point, uppercase letters indicate significant differences (*P* < 0.05) between different nitrogen treatments within the same Cultivars, while lowercase letters indicate significant differences (*P* < 0.05) between cultivars under different nitrogen application levels, based on Duncan's Significant Studentized Range (SSR) test.
