# Supplementary material for: Water phase distribution and its dependence on internal structure in soaking maize kernels: a study using low-field nuclear magnetic resonance and X-ray micro-computed tomography
Source: Front Plant Sci. 2025 Jan 24;15:1529514. doi: 10.3389/fpls.2024.1529514 (PMC11802422; doi:10.3389/fpls.2024.1529514)
Supplement: Supplementary file 2 [file Supplementaryfile2.docx]

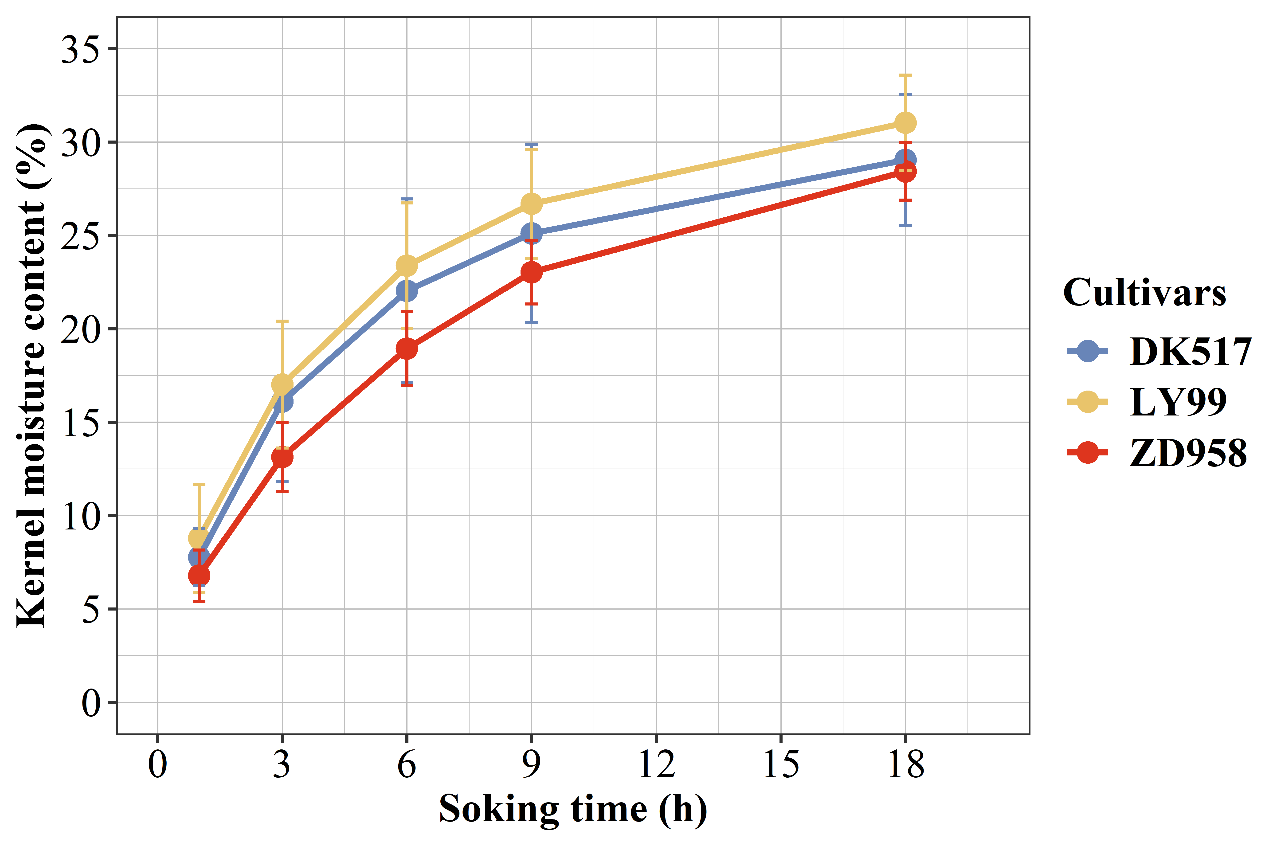


**Supplementary Figure 1.** Response of kernel moisture of different cultivars. The different colored lines represent different cultivars: blue for DK517, yellow for LY99, and red for ZD958.


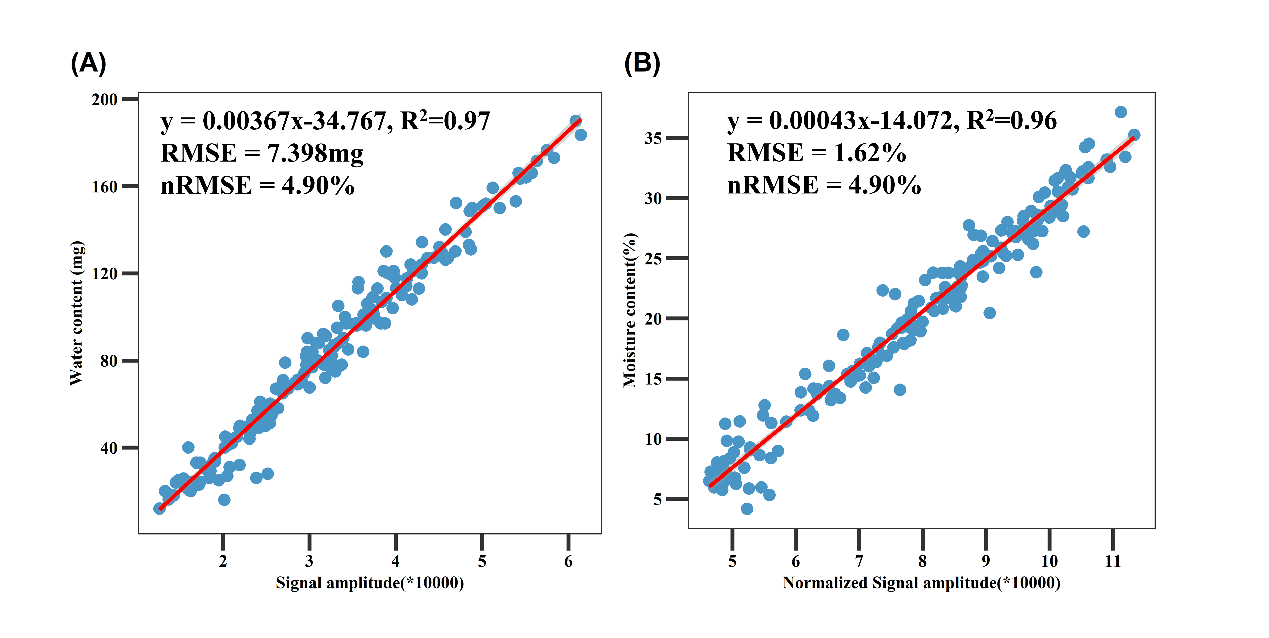


**Supplementary Figure 2.** Correlation between total signal amplitude and water content(A) and moisture content(B)
